# Supplementary material for: Modelling the transmission dynamics of H9N2 avian influenza viruses in a live bird market
Source: Nat Commun. 2024 May 1;15:3494. doi: 10.1038/s41467-024-47703-9 (PMC11063141; doi:10.1038/s41467-024-47703-9)
Supplement: Supplementary file 1 — Supplementary Information [file 41467_2024_47703_MOESM1_ESM.pdf]

Supplementary Information for the manuscript: Modelling the transmission dynamics of H9N2 avian influenza viruses in a live bird market

1 Supplementary Methods

1.1 Additional details on batch initialisation

Let us denote with $P^{(0)}_{g,b,X}$ the probability that a chicken of type b and from group g is in compartment $X \in \{S, E_1, E_2, I, R_+, R_-\}$ at recruitment. In the main text we assumed:

$$\begin{cases} P^{(0)}_{g,b,X} = 1 - \rho_{g,b} \text{ if } X = S \,, \\ P^{(0)}_{g,b,X} = \rho_{g,b} \cdot \bar{P}_X(\sigma_b, \mu, \eta, \lambda_b, \kappa_b) \text{ otherwise } , \end{cases} \tag{S1}$$

where $\rho_{g,b}$ is the probability of prior exposure and $\bar{P}_X$ is the probability of being in compartment $X \in \{E_1, E_2, I, R_+, R_-\}$ conditional on prior exposure, averaged over time since prior exposure.

We first consider the general case where an infected chicken goes through a sequence of n states with transition rates r_k, $k = 1, \ldots, n-1$ from state k to the $k+1$. In this case, the probability $P_k(\tau)$ that the chicken is in state k at time τ evolves according to:

$$\begin{cases} \dot{P}_1(t) = -r_1 P_1 \,, \\ \dot{P}_k(t) = r_{k-1} P_{k-1} - r_k P_k \text{ for } k = 2, \ldots, n-1 \,, \\ \dot{P}_n(t) = r_{n-1} P_{n-1} \,, \end{cases} \tag{S2}$$

with initial conditions $P_k(0) = \delta_{k,1}$, where $\delta_{i,j}$ is Kronecker delta. The dot notation is a shorthand for differentiation with respect to time. The general solution to Eq.(S2) is obtained by integrating equations recursively:

$$\begin{cases} P_1(\tau) = \exp(-r_1 \tau) \,, \\ P_k(\tau) = r_{k-1} \exp(-r_k \tau) \int_0^\tau P_{k-1}(x) \exp(r_k x) dx \text{ for } k = 2, \ldots, n-1 \,, \\ P_n(\tau) = r_{n-1} \int_0^\tau P_{n-1}(x) dx \,. \end{cases} \tag{S3}$$

The explicit solution to Eq.(S3) in the case of our SEEIRR model is:

$$\begin{cases} P_{E_1}(\tau) = \exp\left(-2\sigma\tau\right) , \\ P_{E_2}(\tau) = 2\sigma\tau \exp\left(-2\sigma\tau\right) , \\ P_I(\tau) = \left(\dfrac{2\sigma}{2\sigma-\mu}\right)^2 \left[\exp\left(-\mu\tau\right) - \exp\left(-2\sigma\tau\right)(1+(2\sigma-\mu)\tau)\right] , \\ P_{R_+}(\tau) = \mu\left(\dfrac{2\sigma}{2\sigma-\mu}\right)^2 \left[\exp\left(-\eta\tau\right)\dfrac{(2\sigma-\mu)^2}{(\mu-\eta)(2\sigma-\eta)^2} - \right. \\ \qquad \left. - \dfrac{exp(-\mu\tau)}{\mu-\eta} + \dfrac{\exp\left(-2\sigma\tau\right)}{2\sigma-\eta}\left[\dfrac{4\sigma-\mu-\eta}{2\sigma-\eta} + (2\sigma-\mu)\tau\right]\right] , \\ P_{R_-}(\tau) = 1 - P_{E_1}(\tau) - P_{E_2}(\tau) - P_I(\tau) - P_{R_+}(\tau) . \end{cases} \tag{S4}$$

We now average $P_X(\tau)$ over τ, under the assumption that the latter follows a gamma distribution $f(\tau|\lambda,\kappa)$ with inverse scale λ and shape κ. Mathematically, the average probability $\bar{P}_X$ for compartment X, is computed as $\bar{P}_X = \int_0^\infty P_X(\tau) f(\tau|\lambda,\kappa) d\tau$. In our SEEIRR model:

$$\begin{cases} \bar{P}_{E_1} = \left(\dfrac{\lambda}{\lambda+2\sigma}\right)^\kappa , \\ \bar{P}_{E_2} = \dfrac{2\sigma\kappa\lambda^\kappa}{(\lambda+2\sigma)^\kappa} , \\ \bar{P}_I = \dfrac{4\sigma^2\lambda^\kappa}{(2\sigma-\mu)^2}\left[\dfrac{1}{(\lambda+\mu)^\kappa} - \dfrac{\lambda-\kappa\mu+2(1+\kappa)\sigma}{(\lambda+2\sigma)^{\kappa+1}}\right] , \\ \bar{P}_{R_+} = \dfrac{4\mu\sigma^2\lambda^\kappa}{(2\sigma-\mu)^2}\left[\dfrac{(2\sigma-\mu)(2\sigma+\mu-2\eta)}{(\lambda+\eta)^\kappa(\mu-\eta)(2\sigma-\eta)^2} - \dfrac{1}{(\lambda+\mu)^\kappa(\mu-\eta)} + \right. \\ \qquad \left. + \dfrac{\lambda+2\sigma+\kappa(2\sigma-\mu)(2\sigma-\eta)+(2\sigma-\eta)(2\sigma+\lambda)}{(\lambda+2\sigma)^{\kappa+1}(2\sigma-\eta)^2}\right] , \\ \bar{P}_{R_-} = 1 - \bar{P}_{E_1} - \bar{P}_{E_2} - \bar{P}_I - \bar{P}_{R_+} . \end{cases} \tag{S5}$$

Finally, the statistics of time since past exposure differs between intervention and control chickens because of the delay in recruitment: if $f_c(\tau|\lambda,\kappa) = f(\tau|\lambda,\kappa)$ denotes the distribution of past exposure time for control chickens (i.e. since T_1), the corresponding distribution for intervention chickens is:

$$f_i(\tau|\lambda,\kappa,\delta t = T_1 - T_0) = f(\tau + T_1 - T_0|\lambda,\kappa) / \left[1 - F(T_1 - T_0|\lambda,\kappa)\right] , \tag{S6}$$

where $F(x|\lambda,\kappa) = \int_0^x f(u|\lambda,\kappa)du$. This follows from the assumption that intervention and control chickens share the same distribution, but the former can not be infected between T_0 and T_1, hence the condition $\tau > \delta t$; in addition, we must also shift τ by δt since we are now interested in time elapsed since exposure and T_0, not T_1.

Note that $f(\tau|\lambda,\kappa)$ denotes the probability density of time since past exposure without reference to which compartment a chicken ends up in at $t = 0$. Instead, the probability density $f(\tau|X,\lambda,\kappa)$ that a chicken was infected a time τ in the past, given it is in compartment X at $t = 0$, is given by Bayes' theorem:

$$f(\tau|X,\lambda,\kappa) = \frac{P_X(\tau) f(\tau|\lambda,\kappa)}{\bar{P}_X} \,. \tag{S7}$$

This equation suggests a sampling importance re-sampling scheme to sample τ, conditional on compartment X: first, draw a sample $\tau_k, k = 1, \ldots, n$ from the unconditional distribution $f(\tau|\lambda,\kappa)$ and compute a weight $w_k = P_X(\tau_k)/\bar{P}_X$; second, sample n values with replacement from the set $\{\tau_k\}$ using $w_k/\sum_k w_k$ as re-sampling probabilities. If one is interested in sampling from $f(\tau|X_1 \cup X_2, \lambda, \kappa)$, where X_1, X_2 are distinct compartments, it is possible to use the same procedure after noting that probabilities P_{X_1} and P_{X_2} add up to $P_{X_1 \cup X_2}$.

1.2 Additional details on inference procedure

This section contains further details on the choice of priors (listed in Table S1), and the likelihood function.

We considered a flat distribution for introduction probabilities $\rho_{g,b}$ and the positivity waning rate η and a $\chi^2(4)$ distribution for hyper-parameters λ_{BR}, λ_{BY}, κ_{BR} and κ_{BY} tuning the timing of past infections. Our experimental setup did not allow us to record time to recovery, hence we do not expect our data to be informative about the infectious period. In addition, experimental studies usually report the duration of shedding since the point of inoculation/infection. For these reasons, we decided to set a tight normal prior on the total time from exposure to recovery $T_{EI} = T_I + 0.5(T_{E,BR} + T_{E,BY})$. We did enforce $T_I > 0.5\,d$ and $T_R > 1d$. Finally, we penalized parameter configurations with a large $\beta/\mu l_\beta$ ratio, with $l_\beta > 0$ being an hyper-parameter: the smaller l_β, the stronger the penalty on β.

In order to define the model likelihood, we first denote with $p^+_{g,b}(j)$, $j = 0, 1, 2, 3, 4$ the probability that a chicken of type b and from recruitment group g is positive by T_j for the first time, where the time points T_j are defined in the Materials and Methods section in the main manuscript. Let us also denote the number of chickens of the same type and group becoming positive by T_j for the first time with $n^+_{g,b}(j)$. There are some differences between control and intervention groups in terms of how these counts are performed. First, $n^+_{i,b}(0)$ is the number of intervention chickens that are positive at T_0; note that, by design, $n^+_{c,b}(0) = 0$. Second, $n^+_{i,b}(1)$ is the number of intervention chickens that become positive between T_0 and T_1, while $n^+_{c,b}(1)$ is the number of control chickens that are already positive at T_1. $n^+_{g,b}(j)$, $j = 2, 3, 4$ represents the number of chickens that became positive in $[T_{j-1}, T_j]$. Finally, let us denote with $p^-_{g,b} = 1 - \sum_{j=0}^4 p^+_{g,b}(j)$ the probability to escape infection. Conditioned on these probabilities, the model likelihood $\mathcal{L}(D|\theta)$ is multinomial:

$$\mathcal{L}(D|\theta) \propto \prod_{b=BR,BY} \prod_{g=c,i} \left[p^-_{g,b}\right]^{n^-_{g,b}} \prod_{j=0}^{4} \left[p^+_{g,b}(j)\right]^{n^+_{g,b}(j)} \,, \tag{S8}$$

where $n^-_{g,b}$ is the number of chickens that remained negative for the whole experiment. Note that the likelihood above is based on the assumption that recruited chickens can be treated independently; as explained in the main text, this implicitly assumes that recruited chickens do not contribute to transmission, while remaining susceptible to infection due to other chickens at the market.

The likelihood function in Eq. (S8) also assumes that no chicken dies/is culled prematurely in the experiment. In order to include these chickens in our analysis, while also not modelling deaths/culling explicitly, we consider the corresponding data points as bingh right-censored. Hence, a negative chicken that drops out during the j-th time interval, contributes a factor $1 - \sum_{k=0}^{j-1} p^+_{g,b}(k)$ to $\mathcal{L}$. Chickens that die/are culled prematurely but turn positive at some point during the experiment, contribute the same to $\mathcal{L}$ as chickens that are not removed prematurely, and are already counted in $n^+_{g,b}(j)$.

Unfortunately, most of the $p^+_{g,b}(j)$'s are unknown and can not be computed analytically. We hence estimate these probabilities by simulating $N_{rep} = 10^6$ recruited chickens for each combination of recruitment group g and type b. These N_{rep} chickens were split among $M = 10$ independent simulations.

We counted the mean number of chickens $(\hat{n}^+_{g,b}(j))$ that turn positive at different stages of the experiment, which we used to estimate $p^+_{g,b}(j)$:

$$p^+_{g,b}(j) \approx \frac{\hat{n}^+_{g,b}(j)}{N_{rep}},\ j = 0, 1, 2, 3, 4\,. \tag{S9}$$

1.3 Persistence of environmental contamination.

Contaminated faecal material shed by infectious chickens has been shown to retain its transmission potential for a significant amount of time. Nonetheless, available studies highlighted considerable variation in persistence depending on a number of environmental conditions, including temperature, humidity, wet or dry faeces, salinity and pH [1–5]. This suggests that properties of environmental contamination are likely to vary over months and across different types of chickens. In addition, it is possible that different influenza subtypes differ in their ability to persist in the environment, possibly as a consequence of evolutionary mechanisms [2].

Given this heterogeneity, we selected three values of decay rate Θ, namely $\Theta^{-1} = 10, 3, 1$ days, that are representative of a broad range of environmental conditions. In particular, these values roughly correspond to H9N2 AIV persistence in water at temperatures 15, 25 and 35 °C, respectively [2].

2 Supplementary Figures

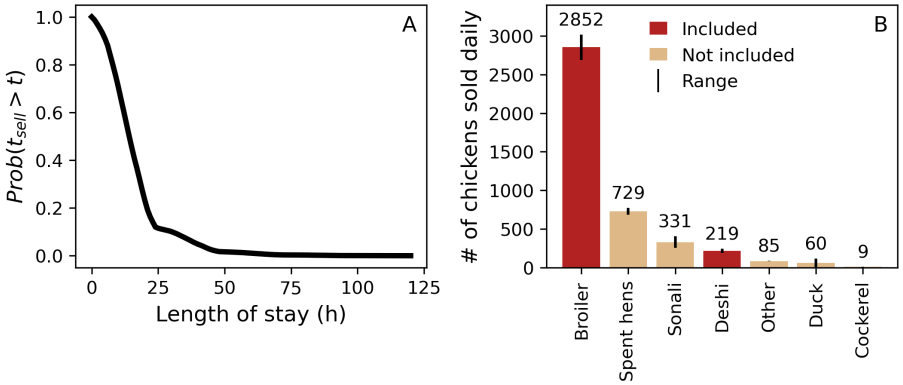

Supplementary Figure 1: **Length of stay distribution and chickens traded daily.** (A) Length of stay survival distribution used in the fit. (B) Bars represent numbers of chickens sold daily at the market (exact numbers are indicated on top of each bar). Black whiskers indicate corresponding ranges, as individual data are given as a range. Mean Broiler and backyard chickens (Deshi) are highlighted in red. Data for both graphs were obtained from [6].

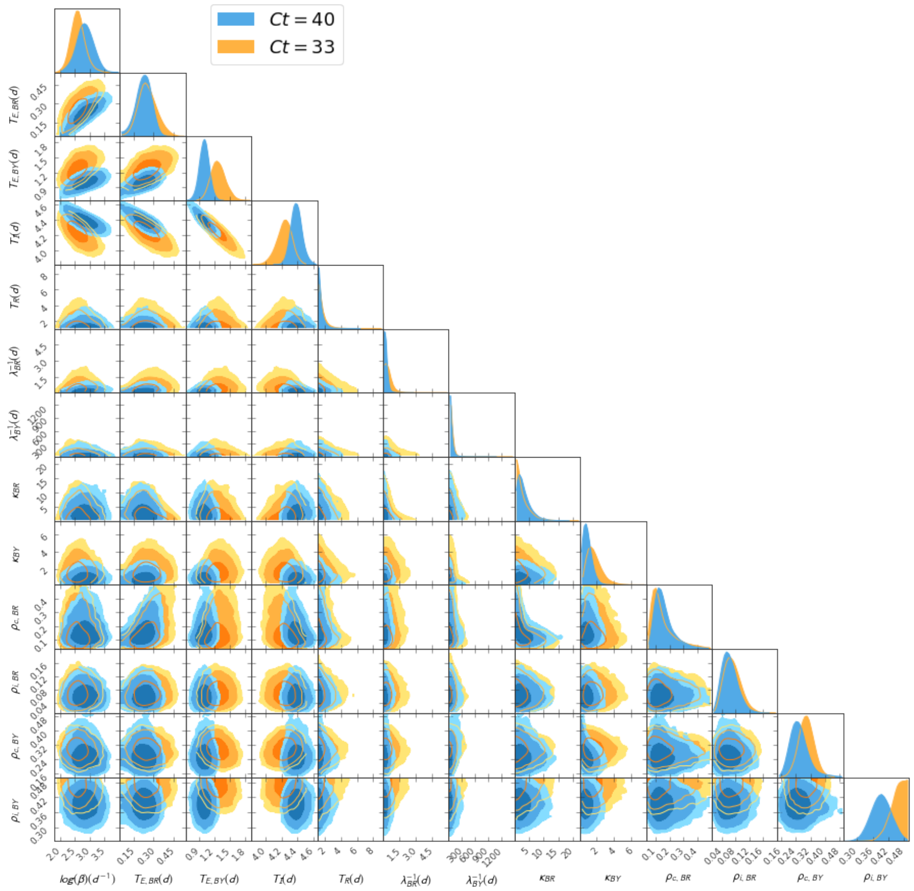

Supplementary Figure 2: **Marginal posterior distributions and pair correlations for fitted parameters.** Diagonal panels show posterior marginal distribution for fits to $Ct = 40$ (blue) and $Ct = 33$ (orange) data. Off-diagonal panels show kernel-smoothed pairwise projections, with 68%, 95% and 99% contour levels. For both fits we set $l_\beta = 0.005$ and $\bar{T}_{EI} = 5$ days. This plot was realised using *pygtc* module, version 0.4.0 [7].

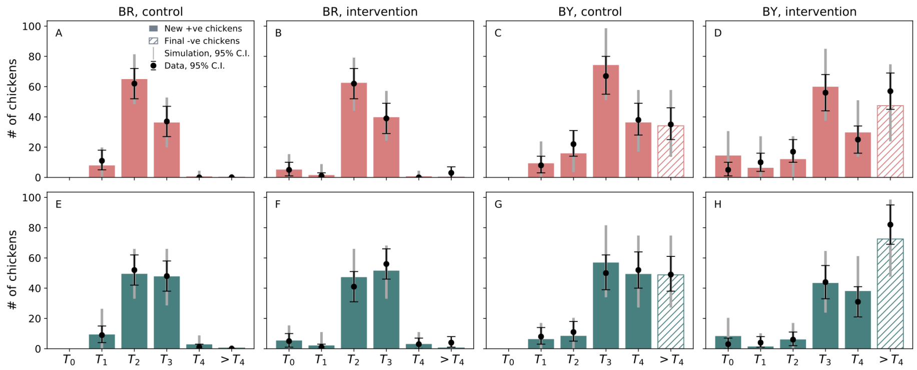

Supplementary Figure 3: **Goodness of fit.** Panels show posterior predictive checks for fits to $Ct = 40$ (A-D) and $Ct = 33$ (E-H) data, broken down by chicken type and recruitment group. Each panel shows posterior mean (bars) and 95% C.I. (grey error bars) for the numbers of chickens becoming positive (filled) at different stages of the experiment or remaining susceptible (hatched). Black dots and error bars denote data and 95% C.I. computed under a binomial distribution assumption. We ran 20000 simulations with 5 control and 5 intervention chickens from 2000 independent posterior samples to estimate the probability of turning positive at different times. Expected counts were obtained by multiplying these probabilities by the number of recruited chickens in each category.

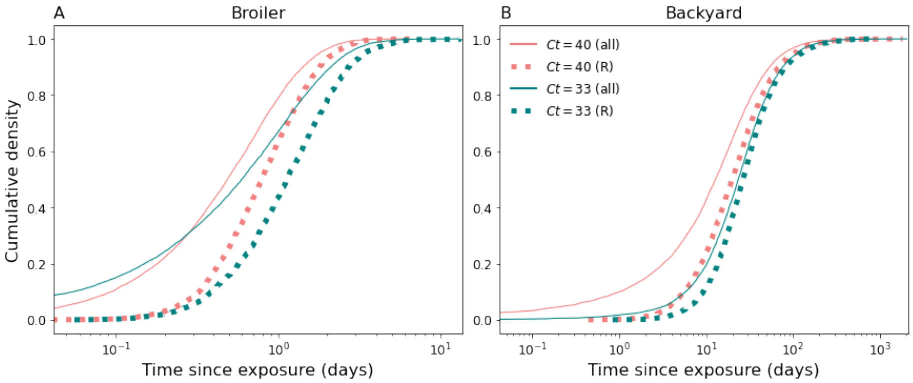

Supplementary Figure 4: **Time since prior exposure.** Panels show cumulative distributions of time since prior exposure for BR (A) and BY (B) chickens, respectively. Red and teal lines correspond to distributions obtained from fits to $Ct = 40$ and $Ct = 33$ data, respectively. Solid lines account for any possible state a chicken might be in when it is introduced in the LBM, provided it had been exposed to AIV previously, whereas dashed lines are restricted to recovered (R_+ and R_-) chickens only. In the former case, we sample once from $f(\tau) \sim \Gamma(\lambda_b, \kappa_b)$, $b = BR, BY$ for each available sample from the posterior distribution. In the latter case, we used sampling importance re-sampling to sample from $f(\tau|R_+ \cup R_-)$ (see Eq. (S7)). For both fits we set $l_\beta = 0.005$ and $\bar{T}_{EI} = 5$ days.

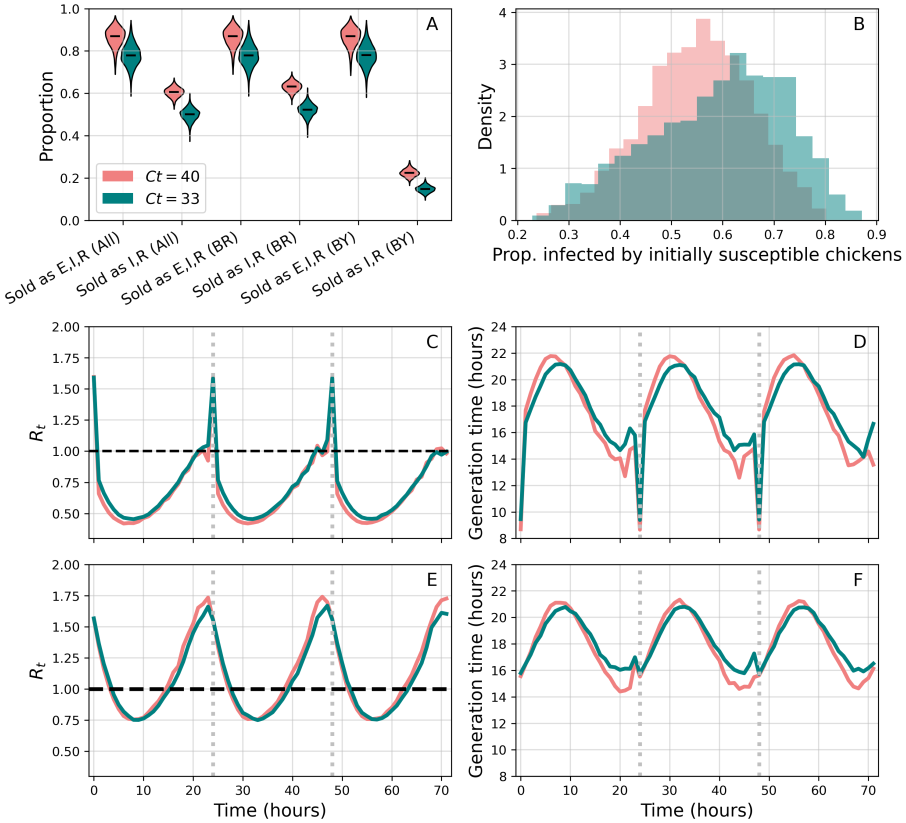

Supplementary Figure 5: **Relative importance of external introductions and local transmission.** (A) Violin plots denote posterior distributions for the fraction of initially susceptible chickens that are infected locally and the fraction of those that become infectious before being sold. (B) Posterior distribution for the fraction of LBM-acquired infections that are caused by other chickens that became infected within the LBM, as opposed to externally-introduced infections. Results are based on 20000 simulations from 2000 independent samples from the posterior distribution. (C) Mean number of new cases (R_t) caused by chickens infected at time t. (D) Mean generation time versus the timing of primary infection t. The generation time is defined as the delay between the infection of a primary and a secondary case. Statistics in C,D were obtained by tracking transmission pairs in an individual-based version of our model. Panels E,F mirror C,D but assume that all chickens entering the market after 30 days are susceptible (i.e. in absence of external introductions). We consider all primary cases infected between $t = 45$ and $t = 48$ days. For externally-introduced infections, the infection time was set to the time of introduction. Daily shipments of chickens are denoted with vertical lines. Results in C-F based on 2000 simulations using the same number of independent samples from the posterior distribution.

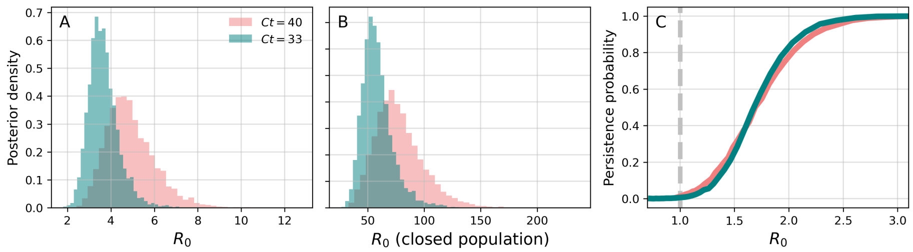

Supplementary Figure 6: **Basic reproductive number and AIV persistence.** (A) Posterior distribution for the basic reproductive number R_0 for fits to $Ct = 40$ (red) and $Ct = 33$ (teal). The latter is calculated as the basic reproductive number for a continuous time $SEEIRR$ model with a constant rate of chicken removal ν, where ν is set to the inverse mean length of stay. Combining previous theoretical results yields the expression $R_0 = \sum_b \left(\frac{2\sigma_b}{2\sigma_b + \nu}\right)^2 \cdot \frac{\bar{N}_b}{N} \cdot \frac{\beta}{\mu + \nu}$ [8–10], where $\bar{N}_b$ is the mean number of chickens of type b present in the LBM, and is estimated through simulations, and $N = \sum_b N_b$ is the number of chickens introduced daily. (B) Posterior distribution for the ratio β/μ, which represents the basic reproductive number in a closed population ($\nu = 0$) of constant size N. (C) Posterior probability of AIV persistence as a function of R_0. This is measured as the proportion of 2000 simulations where at least one latent or infectious chicken is observed at $t = 50$ days, assuming that all chickens entering the market after $t = 20$ days are susceptible. Results in A,B are based on 5000 posterior samples.

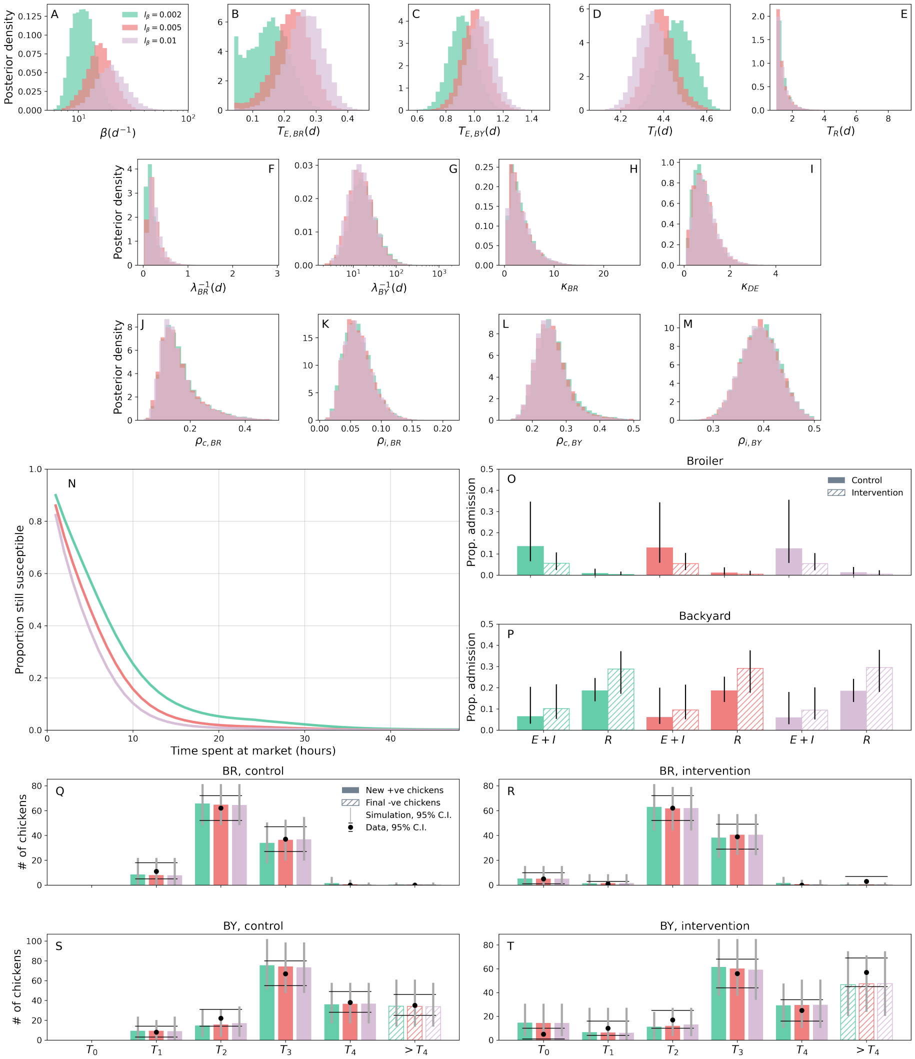

Supplementary Figure 7: **Sensitivity to prior assumptions on β.** Here we repeated the fit to $Ct = 40$ data whilst varying l_β. (A-M) Posterior marginal distributions of fitted parameters. (N) Probability of a chicken remaining susceptible after a given amount of time spent at the market. Probability of broiler (O) and backyard chickens (P) being already in E or I compartments (filled) or recovered (hatched) at market entrance. (Q-T) Posterior predictive checks, realised as in Supplementary Fig. 3 and based on 30000 simulations with 5 control and 5 intervention chickens from 3000 independent posterior samples. We set $\bar{T}_{EI} = 5\,d$ for all fits.

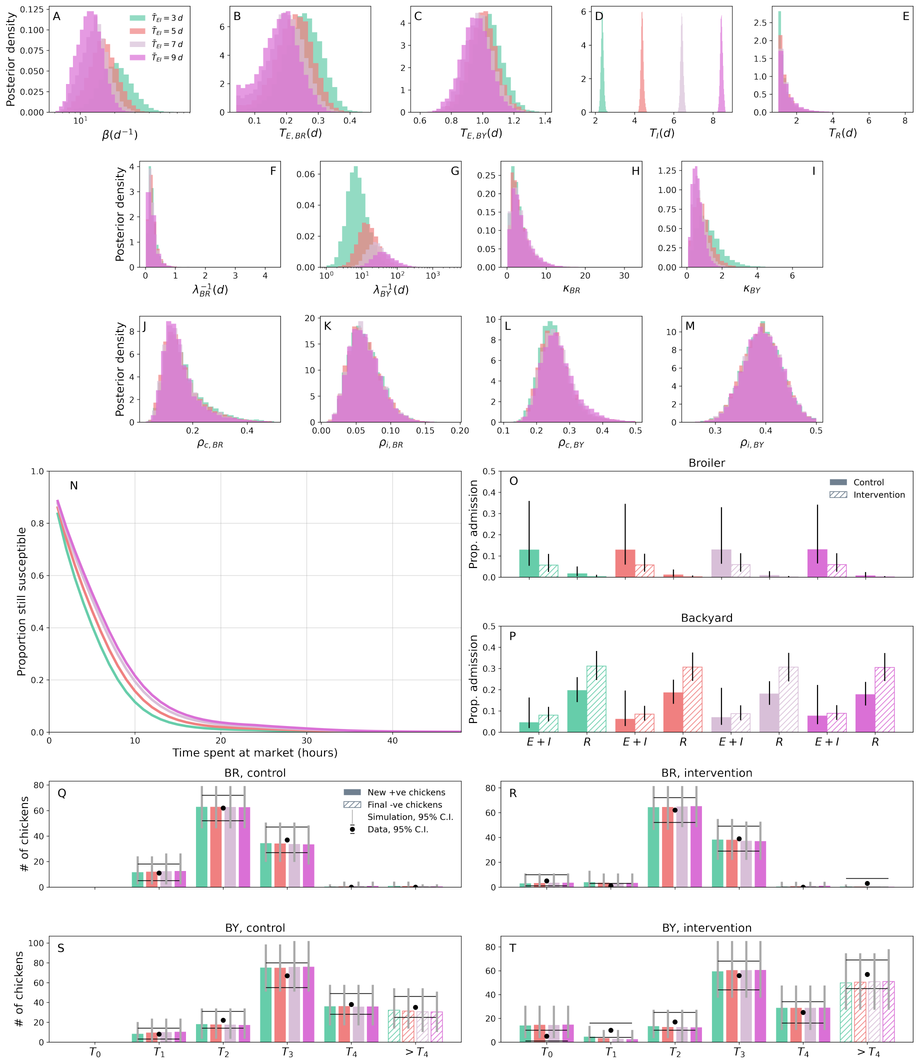

Supplementary Figure 8: **Sensitivity to prior assumptions on $\bar{T}_{EI}$.** Here we repeat the fit to $Ct = 40$ data whilst varying the hyper-parameter $\bar{T}_{EI}$. Figure structure is the same as Fig. S7. We set $l_\beta = 0.005$ for all fits.

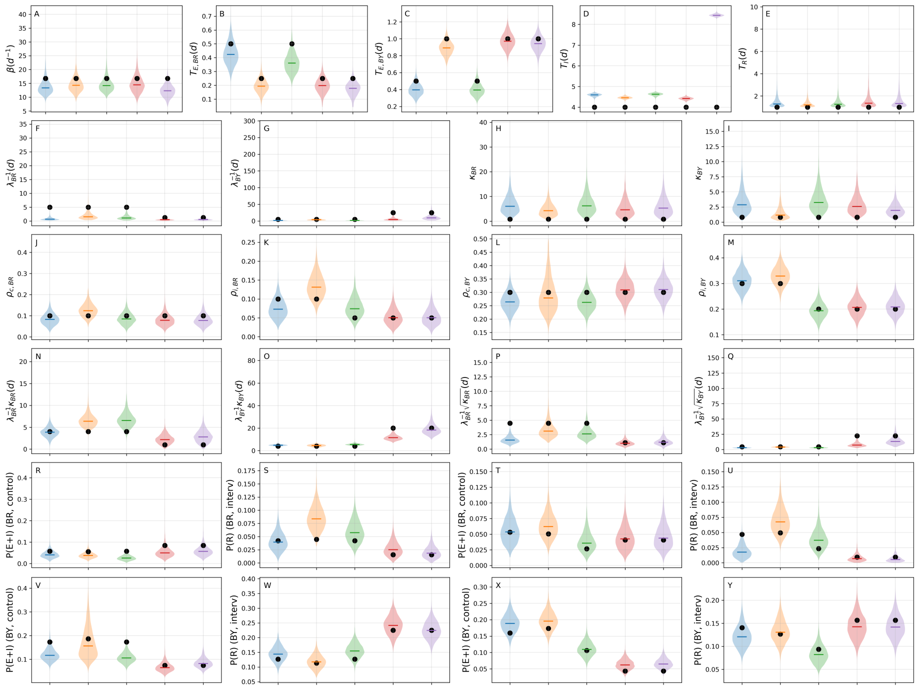

Supplementary Figure 9: **Model fit to simulated data.** We used our model to generate synthetic data under 5 different parameter sets. The model was then fitted to these data and its ability to recover the initial parameters was assessed. Parameter values in each scenario were selected to reflect multiple types of heterogeneity across chicken types and recruitment groups. In the baseline scenario, the leftmost in each panel, model parameters are the same for broiler and backyard chickens (true parameter values are indicated with black markers). Scenarios 2 and 3 introduce differences in latent periods and $\rho_{g,b}$ across chicken types, respectively, while scenario 4 accommodates both variations simultaneously. Scenario 5 is identical to 4, but the prior distribution on total shedding time is strongly misspecified ($\bar{T}_{EI} = 9$ days instead of 5 days in previous fits). For each parameter set, we simulated 100 independent datasets of the same size as the empirical one; then, we selected the sample that deviated the least from the average trajectory $\bar{n}_{g,b}^{+/-}(t)$, based on the L_2 distance. Panels show posterior marginal distributions (violin plots) for fitted parameters, mean and standard deviation of type-specific time since past infection-given by $\lambda_b^{-1}\kappa_b$ and $\lambda_b^{-1}\sqrt{\kappa_b}$, respectively, and probabilities of chickens entering the market as latent or infectious, $P(E+I)$, and recovered, $P(R)$. The mismatch in the estimate of T_I in scenario 5 (D) is due to the misspecified prior on shedding time, and the fact that the data is more informative about T_E (B,C) than T_I . For each fit, we obtained 3080 samples (using the same thinning and burn-in as in the main analysis). In all fits, we set $l_\beta = 0.005$.

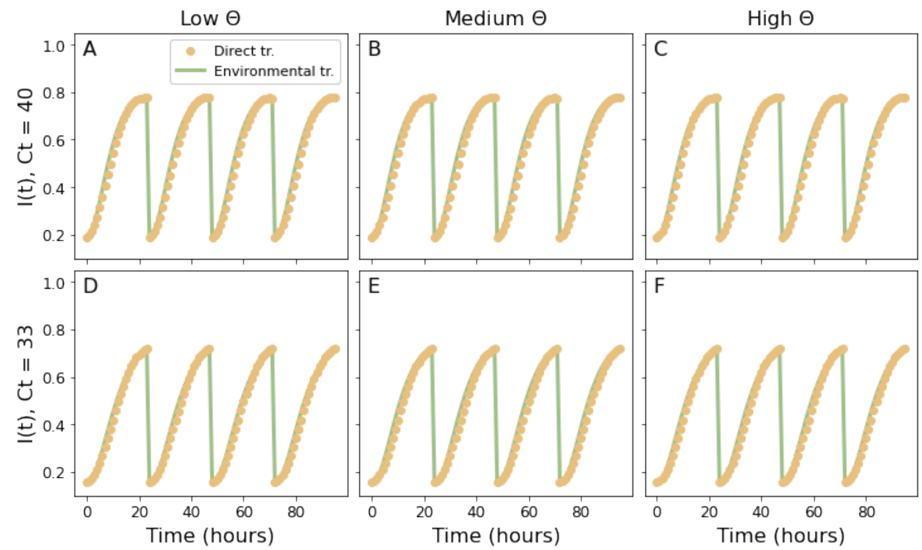

Supplementary Figure 10: **Comparison of direct and environmental transmission.** Each panel shows average AIV prevalence in chickens under direct (dots) and environmental transmission (line) for three different values of decay rate Θ corresponding to different columns. First and second rows correspond to posterior samples from fits to $Ct = 40$ and $Ct = 33$ data, respectively. In both cases, we ran 5000 simulations from 500 posterior samples. In the case of environmental transmission, we mapped β to $\beta_{env} = \beta \cdot (1 - e^{-\Theta})$ for each posterior sample.

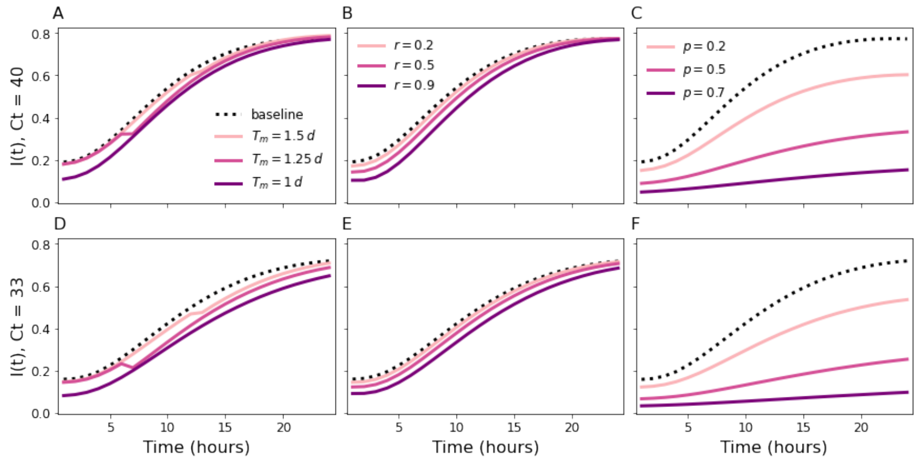

Supplementary Figure 11: **Prevalence dynamics under intervention measures and direct transmission.** Results for early chicken removal/culling (A,D), reduction in probability of prior exposure (B,E) and preemptive immunisation through vaccination (C,F). Each panel shows the time series of AIV prevalence over a single day, under varying levels of strength of intervention. Top and bottom rows correspond to posterior samples from fits to $Ct = 40$ and $Ct = 33$ data, respectively. In both cases, we ran 5000 simulations from 500 posterior samples.

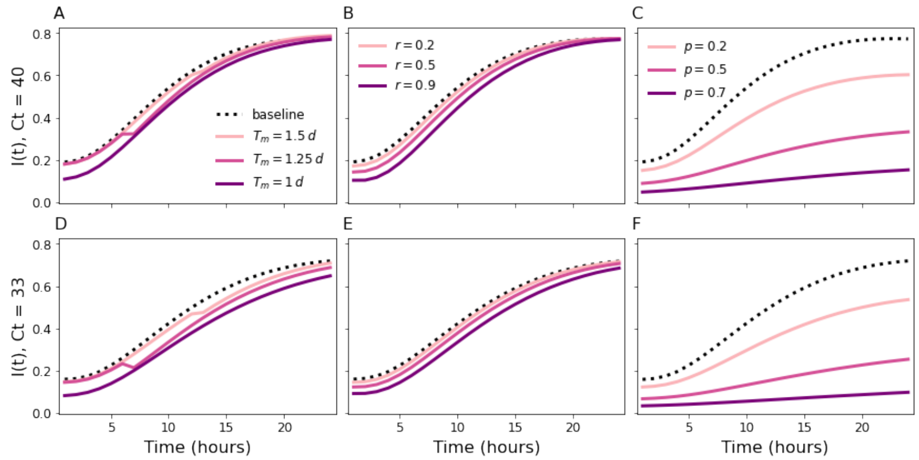

Supplementary Figure 12: **Prevalence dynamics under intervention measures and environmental transmission.** Results for early chicken removal/culling (A,D), reduction in probability of prior exposure (B,E) and preemptive immunisation through vaccination (C,F). Each panel shows the time series of AIV prevalence over a single day, under varying levels of strength of intervention. Top and bottom rows correspond to posterior samples from fits to $Ct = 40$ and $Ct = 33$ data, respectively. In both cases, we ran 5000 simulations from 500 posterior samples. We mapped β to $\beta_{env} = \beta \cdot (1 - e^{-\Theta})$ for each posterior sample. We used the same value of Θ as in the main text.

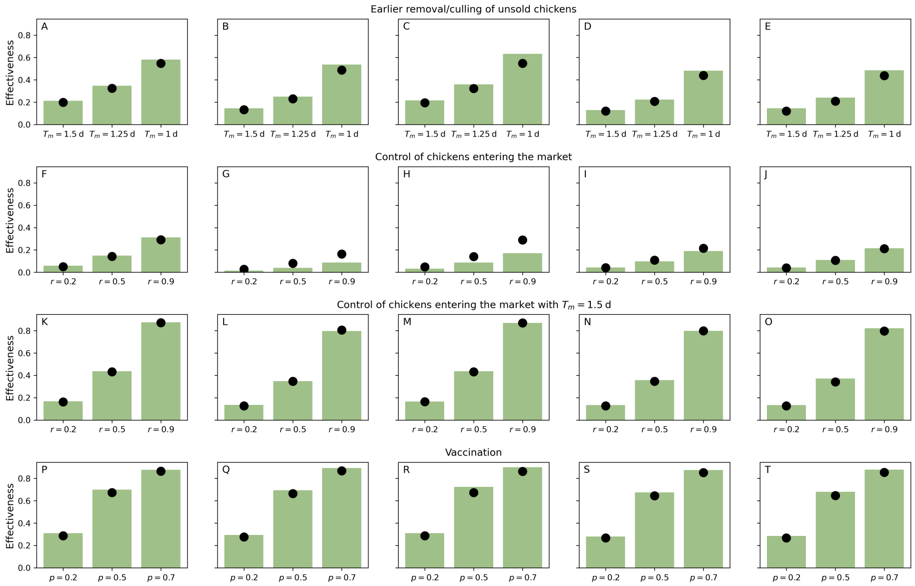

Supplementary Figure 13: **Estimating effectiveness of interventions in artificial scenarios.** This figure compares the effectiveness of veterinary public health interventions calculated using recovered (bars) and original (dots) parameters in the 5 artificial scenarios considered in Supplementary Fig. 9 (from left to right). We consider the same interventions presented in Fig. 2 in the main manuscript: shorter length of stay (top row), control of infected chickens entering the LBM (second row) and vaccination (bottom row). The third row corresponds to the simultaneous implementation of the first two interventions. Moderate bias in posterior estimates of model parameters (Supplementary Fig. 9) does not alter our conclusions about the impact of interventions. Panels G,H indicate an underestimation of the impact of controlling incoming infected birds, which we ascribe to overestimating $\rho_{c,b}$ in artificial scenarios 2 and 3. Results are based on 1000 and 5000 simulations for original and recovered parameters, respectively. In the second case, simulations are evenly split across 500 independent samples from the recovered posterior distribution.

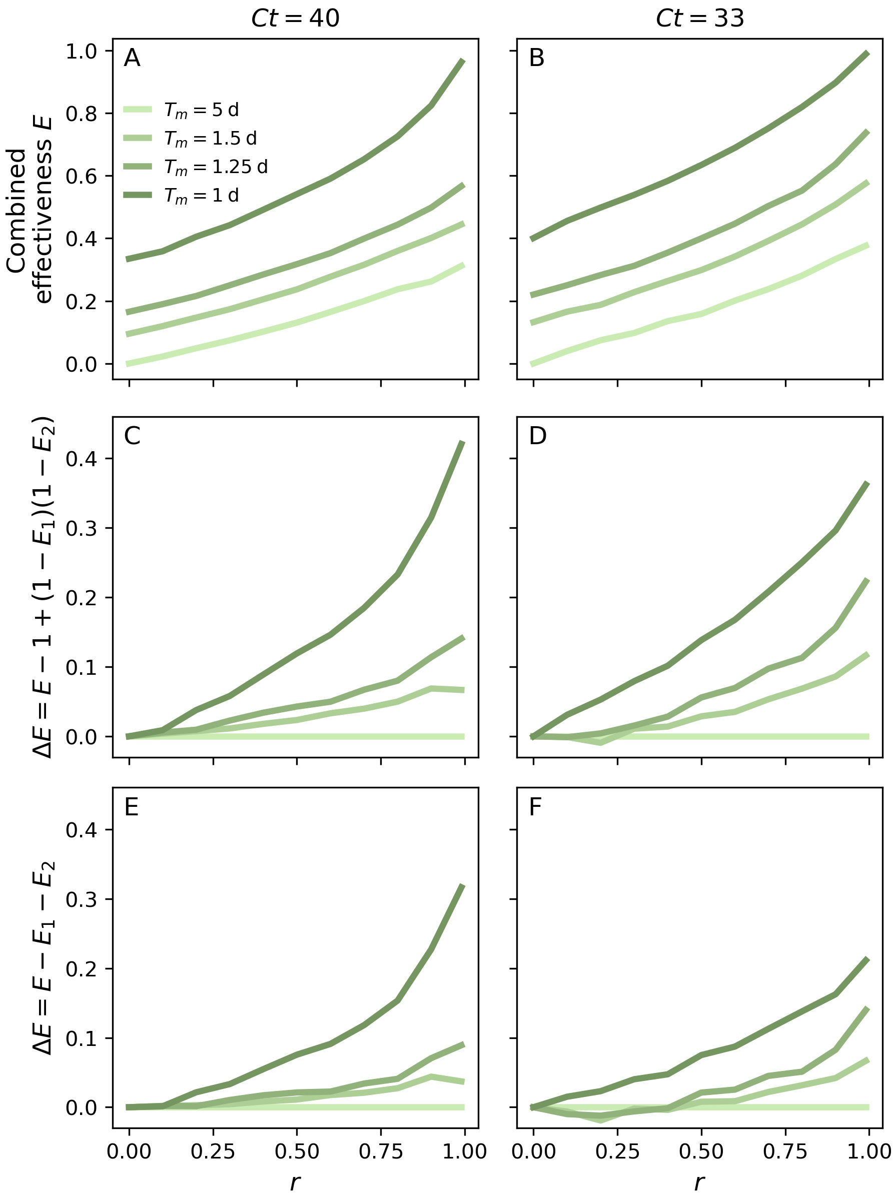

Supplementary Figure 14: **Synergistic effects of combining interventions.** This figure considers a simultaneous reduction of the maximum length of stay of chickens to T_m and the probability of prior exposure $\rho_{c,b}$ to $(1-r)\rho_{c,b}$ (A,B). The effectiveness of these interventions combined is denoted with E, and is defined as the reduction in cumulative daily prevalence relative to a scenario with no intervention in place. The latter corresponds to $T_m = 5$ days and $r = 0$. The effectiveness of individual interventions is denoted as E_1 ($T_m < 5$ days and $r = 0$) and E_2 ($T_m = 5$ days and $0 < r \leq 1$). Panels C-F assess whether the effects of individual interventions add up in a multiplicative (C,D) or additive fashion (E,F). If the effect was purely multiplicative, the expected, combined effectiveness would be $\bar{E} = 1 - (1 - E_1)(1 - E_2)$. In the purely additive case it would be $\bar{E} = E_1 + E_2$. C-F show that $E > \bar{E}$ for most values of T_m and r, suggesting a synergistic effect of implementing these interventions simultaneously. Results are based on 2000 simulations from 200 independent samples from the posterior distribution. The first and second columns correspond to the fits to $Ct = 40$ and $Ct = 33$ data, respectively.

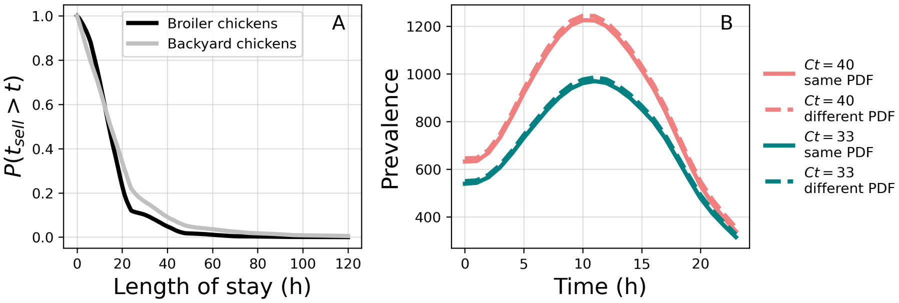

Supplementary Figure 15: **Epidemic dynamics with different length of stay distributions.** (A) Length of stay survival distributions for broiler (black) and backyard (grey) chickens. (B) Average hourly number of infectious chickens when using a shared (solid) or a type-specific (dashed) length of stay distribution. Red and teal lines correspond to fits to $Ct = 40$ and $Ct = 33$ data, respectively. In both cases, we ran 5000 simulations from 500 posterior samples obtained under a shared length of stay distribution (as in the main analysis).

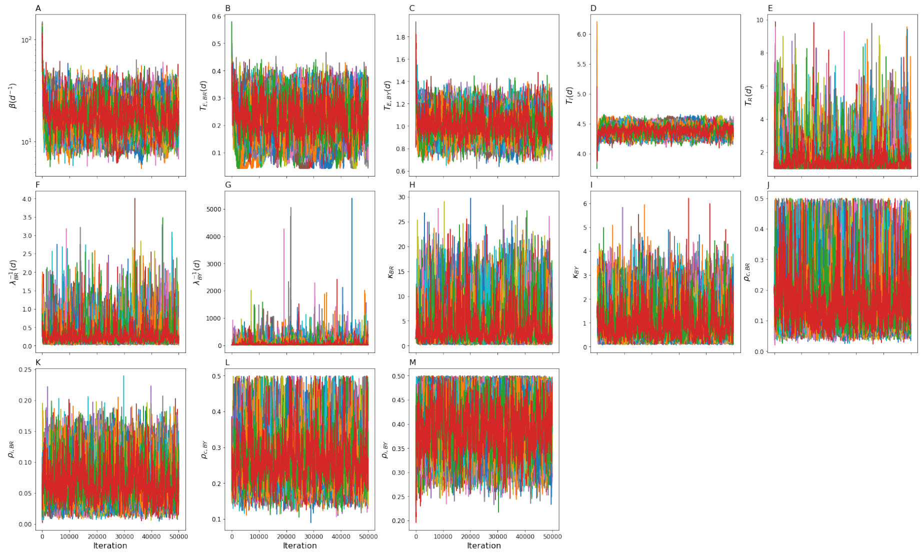

Supplementary Figure 16: **Trace plot for fitted parameters.** Each panel shows the dynamics of individual Markov chains obtained from the fit to $Ct = 40$ data, up to iteration 50000. For this fit we set $l_\beta = 0.005$ and $\bar{T}_{EI} = 5$ days.

3 Supplementary Tables

Supplementary Table 1: **Fitted parameters and priors.** We set a tight normal prior on $T_{EI} = \mu^{-1} + (\sigma_{BR}^{-1} + \sigma_{BY}^{-1})/2$, with mean $\bar{T}_{EI}$. The prior on β assigns a penalty to values that are too large compared to $N\mu l_\beta$, where N is the total number of chickens introduced on a given day and l_β is an hyper-parameter. Baseline values for hyper-parameters are indicated in bold.

Name	Prior	Range	Notes
β	$\propto \mu^{-1} e^{-\beta/N\mu l_\beta}$	$(0, \infty)$	$l_\beta = 0.002, \mathbf{0.005}, 0.1$
σ_{BR}	$T_{EI} \sim \mathcal{N}(\bar{T}_{EI}, 6.6 \cdot 10^{-3}\, d)$	$(0.05, 24)\, d^{-1}$	$\bar{T}_{EI} = 3, \mathbf{5}, 7, 9\, d$
σ_{BY}	$T_{EI} \sim \mathcal{N}(\bar{T}_{EI}, 6.6 \cdot 10^{-3}\, d)$	$(0.05, 24)\, d^{-1}$	See above
μ	$T_{EI} \sim \mathcal{N}(\bar{T}_{EI}, 6.6 \cdot 10^{-3}\, d)$	$(0.067, 0.5)\, d^{-1}$	See above
η	Flat	$(0.1, 1)\, d^{-1}$	-
λ_{BR}	$\chi^2(4)$	$(0, \infty)\, d^{-1}$	-
λ_{BY}	$\chi^2(4)$	$(0, \infty)\, d^{-1}$	-
κ_{BR}	$\chi^2(4)$	$(0, \infty)$	-
κ_{BY}	$\chi^2(4)$	$(0, \infty)$	-
$\rho_{c,BR}$	Flat	$(0, 0.5]$	-
$\rho_{i,BY}$	Flat	$(0, 0.5]$	-
$\rho_{c,BR}$	Flat	$(0, 0.5]$	-
$\rho_{i,BY}$	Flat	$(0, 0.5]$	-

References

1. Dalziel, A. E., Delean, S., Heinrich, S. & Cassey, P. Persistence of Low Pathogenic Influenza A Virus in Water: A Systematic Review and Quantitative Meta-Analysis. *PLOS ONE* **11,** e0161929 (2016).
2. Handel, A., Brown, J., Stallknecht, D. & Rohani, P. A Multi-scale Analysis of Influenza A Virus Fitness Trade-offs due to Temperature-dependent Virus Persistence. *PLOS Computational Biology* **9,** e1002989 (2013).
3. Kormuth, K. A. *et al.* Environmental Persistence of Influenza Viruses Is Dependent upon Virus Type and Host Origin. *mSphere* **4,** e00552–19 (2019).
4. Kurmi, B. *et al.* Survivability of Highly Pathogenic Avian Influenza H5N1 Virus in Poultry Faeces at Different Temperatures. *Indian Journal of Virology* **24,** 272–277 (2013).
5. Shortridge, K. F. *et al.* Characterization of Avian H5N1 Influenza Viruses from Poultry in Hong Kong. *Virology* **252,** 331–342 (1998).
6. Moyen, N. *et al.* Avian influenza transmission risk along live poultry trading networks in Bangladesh. *Scientific Reports* **11,** 19962 (2021).

7. Bocquet, S. & Carter, F. W. pygtc: beautiful parameter covariance plots (aka. Giant Triangle Confusograms). *Journal of Open Source Software* **1,** 46 (2016).
8. d'Onofrio, A. Stability properties of pulse vaccination strategy in SEIR epidemic model. *Mathematical Biosciences* **179,** 57–72 (2002).
9. Diekmann, O., Heesterbeek, J. a. P. & Roberts, M. G. The construction of next-generation matrices for compartmental epidemic models. *Journal of the Royal Society, Interface* **7,** 873–885 (2010).
10. Champredon, D., Dushoff, J. & Earn, D. J. D. Equivalence of the Erlang-Distributed SEIR Epidemic Model and the Renewal Equation. *SIAM Journal on Applied Mathematics* **78,** 3258–3278 (2018).
