# Supplementary material for: Modelling the transmission dynamics of H9N2 avian influenza viruses in a live bird market
Source: Nat Commun. 2024 May 1;15:3494. doi: 10.1038/s41467-024-47703-9 (PMC11063141; doi:10.1038/s41467-024-47703-9)
Supplement: Supplementary file 3 — Reporting Summary [file 41467_2024_47703_MOESM3_ESM.pdf]

nature portfolio

Corresponding author(s): Francesco Pinotti

Last updated by author(s): Mar 27, 2024

Reporting Summary

Nature Portfolio wishes to improve the reproducibility of the work that we publish. This form provides structure for consistency and transparency in reporting. For further information on Nature Portfolio policies, see our Editorial Policies and the Editorial Policy Checklist.

Statistics

For all statistical analyses, confirm that the following items are present in the figure legend, table legend, main text, or Methods section.

n/a	Confirmed	
☐	☒	The exact sample size (n) for each experimental group/condition, given as a discrete number and unit of measurement
☐	☒	A statement on whether measurements were taken from distinct samples or whether the same sample was measured repeatedly
☒	☐	The statistical test(s) used AND whether they are one- or two-sided<br>*Only common tests should be described solely by name; describe more complex techniques in the Methods section.*
☒	☐	A description of all covariates tested
☒	☐	A description of any assumptions or corrections, such as tests of normality and adjustment for multiple comparisons
☐	☒	A full description of the statistical parameters including central tendency (e.g. means) or other basic estimates (e.g. regression coefficient) AND variation (e.g. standard deviation) or associated estimates of uncertainty (e.g. confidence intervals)
☒	☐	For null hypothesis testing, the test statistic (e.g. F, t, r) with confidence intervals, effect sizes, degrees of freedom and P value noted<br>*Give P values as exact values whenever suitable.*
☐	☒	For Bayesian analysis, information on the choice of priors and Markov chain Monte Carlo settings
☒	☐	For hierarchical and complex designs, identification of the appropriate level for tests and full reporting of outcomes
☒	☐	Estimates of effect sizes (e.g. Cohen's d, Pearson's r), indicating how they were calculated

Our web collection on statistics for biologists contains articles on many of the points above.

Software and code

Policy information about availability of computer code

Data collection	No data collection was undertaken in this study.
Data analysis	We used Python (v3.8) to analyze the data. We performed Bayesian inference using the module emcee v3.1.1. Marginal posterior distributions were visualized using the module pygtc v0.4.0. Raw data and companion code are available on Zenodo (https://zenodo.org/records/10886118)

For manuscripts utilizing custom algorithms or software that are central to the research but not yet described in published literature, software must be made available to editors and reviewers. We strongly encourage code deposition in a community repository (e.g. GitHub). See the Nature Portfolio guidelines for submitting code & software for further information.

Data

Policy information about availability of data

All manuscripts must include a data availability statement. This statement should provide the following information, where applicable:

- Accession codes, unique identifiers, or web links for publicly available datasets
- A description of any restrictions on data availability
- For clinical datasets or third party data, please ensure that the statement adheres to our policy

Field experiment data was collected in a previous study (https://www.biorxiv.org/content/10.1101/2023.11.10.566573v1.full.pdf). The raw data can be accessed on Zenodo (https://zenodo.org/records/10886118).

Research involving human participants, their data, or biological material

Policy information about studies with human participants or human data. See also policy information about sex, gender (identity/presentation), and sexual orientation and race, ethnicity and racism.

Reporting on sex and gender	N.a.
Reporting on race, ethnicity, or other socially relevant groupings	N.a.
Population characteristics	N.a.
Recruitment	N.a.
Ethics oversight	N.a.

Note that full information on the approval of the study protocol must also be provided in the manuscript.

Field-specific reporting

Please select the one below that is the best fit for your research. If you are not sure, read the appropriate sections before making your selection.

☐ Life sciences ☐ Behavioural & social sciences ☒ Ecological, evolutionary & environmental sciences

For a reference copy of the document with all sections, see nature.com/documents/nr-reporting-summary-flat.pdf

Ecological, evolutionary & environmental sciences study design

All studies must disclose on these points even when the disclosure is negative.

Study description	We fitted a mechanistic model of avian influenza transmission in a live bird market to data from a field experiment in a Bangladeshi live bird market, with the objective of characterizing avian influenza epidemiology in such setting. We used simulations to assess the effectiveness of a range of veterinary public health interventions aiming to mitigate viral circulation. Quantitative data included counts of chickens testing positive during 64 experimental replicates involving 10 chickens each. Chickens were stratified by chicken type (exotic broiler or backyard chicken) and experimental group (control or intervention).
Research sample	Data consists of counts of chickens testing positive to H9N2 avian influenza virus in a field experiment. 300 exotic broilers and 340 backyard chickens were included in this study. Further details on the research sample can be found at https://www.biorxiv.org/content/10.1101/2023.11.10.566573v1.full.pdf.
Sampling strategy	Data had been collected by collaborators in a previous study. Commercial farms were selected proportionally to estimated amounts of chickens supplied from sub-districts to markets in Chattogram. Villages were selected by generating random coordinates within the study area. Commercial farms with more than 2-3 days left to complete their production cycle were chosen to limit the probability of sampling chickens already infected with avian influenza viruses, as farms' visits by traders were considered as promoting the risk of viral incursion on farms. Chickens showing any clinical sign, including anorexia, oedematous comb or wattles, sneezing, nasal discharge, swollen eyelids or sinuses, heavy breathing, diarrhoea, haemorrhages, or feather loss were excluded from sampling. Sample size was calculated with the objective of yielding enough statistical power (0.8) to detect significant differences (with 0.05 confidence) in the frequencies of positive birds between intervention and control chickens at time T1, i.e. when they were caged together in the market. Sample size was calculated via simulations and assuming a binomial distribution of counts of positive chickens in either experimental group. Further details on sample size calculations can be found at https://www.biorxiv.org/content/10.1101/2023.11.10.566573v1.full.pdf.
Data collection	Data was collected by Lisa Kohnle in a previous study. For each batch, 5 chickens were recruited from mobile traders delivering chickens at the market or from market vendors supplied shortly before (control group), and 5 from commercial farms or villages around Chattogram city (intervention group). Intervention chickens were transported to Chattogram Veterinary and Animal Sciences University and stored in a purpose-build poultry shed. Thorough cleaning of the shed and disinfecting of all surfaces in contact with chickens were carried out to minimise the risk of contamination between different intervention groups and from the environment. After 2.5 days, intervention chickens were transferred to the market in Chattogram, where 5 control chickens were recruited from market stalls. The 10 chickens were then kept in a single cage for 3.5 days. Oropharyngeal swabs were collected from chickens at different time points. Samples were placed in tubes with viral transport medium and sent to the laboratory in a cold box and finally frozen at -80°C. Viral RNA was extracted with MagMAX™-96 Viral RNA Isolation Kits (Thermo Fisher Scientific, Applied Biosystems™) and screened for H9 haemagglutinin gene using real-time reverse transcription PCR (rRT-PCR) with AgPath-IDTM One-Step RT-PCR Reagents (Thermo Fisher Scientific, Applied Biosystems™). Cycle thresholds were finally determined for each sample.
Timing and spatial scale	Data was collected in south-eastern Bangladesh between March and August 2019. Data was collected throughout this period with only a short break for Ramadan in May. Study locations comprised Chattogram Veterinary and Animal Sciences University, production sites in Chattogram and neighbouring districts, and a poultry market in Chattogram city.

Data exclusions	We excluded batches of chickens displaying an unusual number of missing PCR entries and suspected of sample cross-contamination.
Reproducibility	Our study involved only numerical experiments. Relevant code has been provided to ensure reproducibility of numerical simulations and data analysis.
Randomization	Randomization was not possible in this study since control and intervention chickens were recruited at different locations (farms and market stalls, respectively).
Blinding	Blinding was considered unnecessary since it was not expected to affect the rate at which animals in different groups became infected.

Did the study involve field work? ☐ Yes ☒ No

Reporting for specific materials, systems and methods

We require information from authors about some types of materials, experimental systems and methods used in many studies. Here, indicate whether each material, system or method listed is relevant to your study. If you are not sure if a list item applies to your research, read the appropriate section before selecting a response.

Materials & experimental systems

n/a	Involved in the study	
☒	☐	Antibodies
☒	☐	Eukaryotic cell lines
☒	☐	Palaeontology and archaeology
☒	☐	Animals and other organisms
☒	☐	Clinical data
☒	☐	Dual use research of concern
☒	☐	Plants

Methods

n/a	Involved in the study	
☒	☐	ChIP-seq
☒	☐	Flow cytometry
☒	☐	MRI-based neuroimaging
